# Late small bowel perforation from a migrated double plastic biliary stent: A case report and a review of literature of 85 cases from 2000 to 2022

**DOI:** 10.1002/ccr3.7425

**Published:** 2023-11-19

**Authors:** Ibraheem M. Alkhawaldeh, Osama Shattarah, Jehad Feras AlSamhori, Mohammad Abu‐Jeyyab, Abdulqadir J. Nashwan

**Affiliations:** ^1^ School of Medicine Mutah University Al‐Karak Jordan; ^2^ General Surgery Department, School of Medicine Mutah University Al‐Karak Jordan; ^3^ Faculty of Medicine The University of Jordan Amman Jordan; ^4^ Hamad Medical Corporation Doha Qatar

**Keywords:** biliary stent, biliary stent migration, endoscopic retrograde cholangiopancreatography, small bowel perforation

## Abstract

**Key Clinical Message:**

This case highlights the importance of considering stent migration as a possible cause of intestinal perforation and the need for prompt surgical intervention.

**Abstract:**

Endo‐biliary stent displacement is rare but can cause intestinal perforation. An 85‐year‐old woman with a history of ERCPs and biliary stents experienced stomach pain and vomiting. She was diagnosed with small bowel perforation from migrated stents and underwent emergency laparotomy, bowel resection, and tension‐free stapled anastomosis.

## Graphical abstract

Migrated Plastic stent 6 cm in length with 10 fr causing late small bowel perforation.

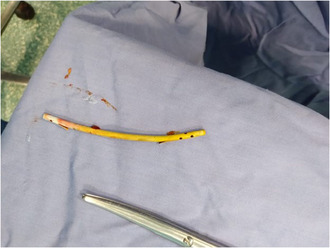

## 1 INTRODUCTION

Internal biliary drainage and endoscopic biliary stents are frequently utilized during endoscopic retrograde cholangiopancreatography (ERCP). It has been used for benign or malignant illnesses and temporary or long‐term biliary system decompression. Based on their material, biliary stents can be classified as either plastic or metallic, with the former being less expensive and simpler to remove or replace.^1^ Stent occlusion from clogging, which may lead to cholecystitis or cholangitis, pancreatitis from duct manipulation, bleeding, stent fracture, and stent migration are complications of biliary stent placement.^2^, ^3^ In addition, there are sporadic cases of endo‐biliary stent displacement and sliding from the common bile duct (CBD).^3^ Although it has a low incidence of <1%, intestinal perforation is a serious potential consequence of stent migration and can happen in any section of the small or large bowel.^4^ A tiny percentage of migrating stents may cause perforation and peritonitis, necessitating an emergency laparotomy, even if most pass silently in the stool or can be removed using endoscopy and fluoroscopy.^5^ This article describes a case of an 85‐year‐old patient who displayed acute abdominal signs and symptoms. A distal small intestinal perforation caused by stent penetration was discovered following an emergency laparotomy. A side‐to‐side tension‐free stapled anastomosis was used to repair the damaged colon.

## 2 CASE PRESENTATION

### 2.1 Chief complaint

Diffuse abdominal pain.

### 2.2 History of present illness

The emergency room at King Abdullah University Hospital had a visit from an 85‐year‐old non‐smoking female patient who is free of any medical conditions and complaining of sharp diffuse stomach pain and several episodes of non‐bloody and non‐bilious vomiting for 3 days.

She described the discomfort as being in the lower abdomen, cramping continuously, and not radiating. It was linked to abdominal bloating. The patient did not report having a fever, jaundice, itching, or a change in the color of their urine or feces.

### 2.3 History of past illness

**FIGURE 1:**
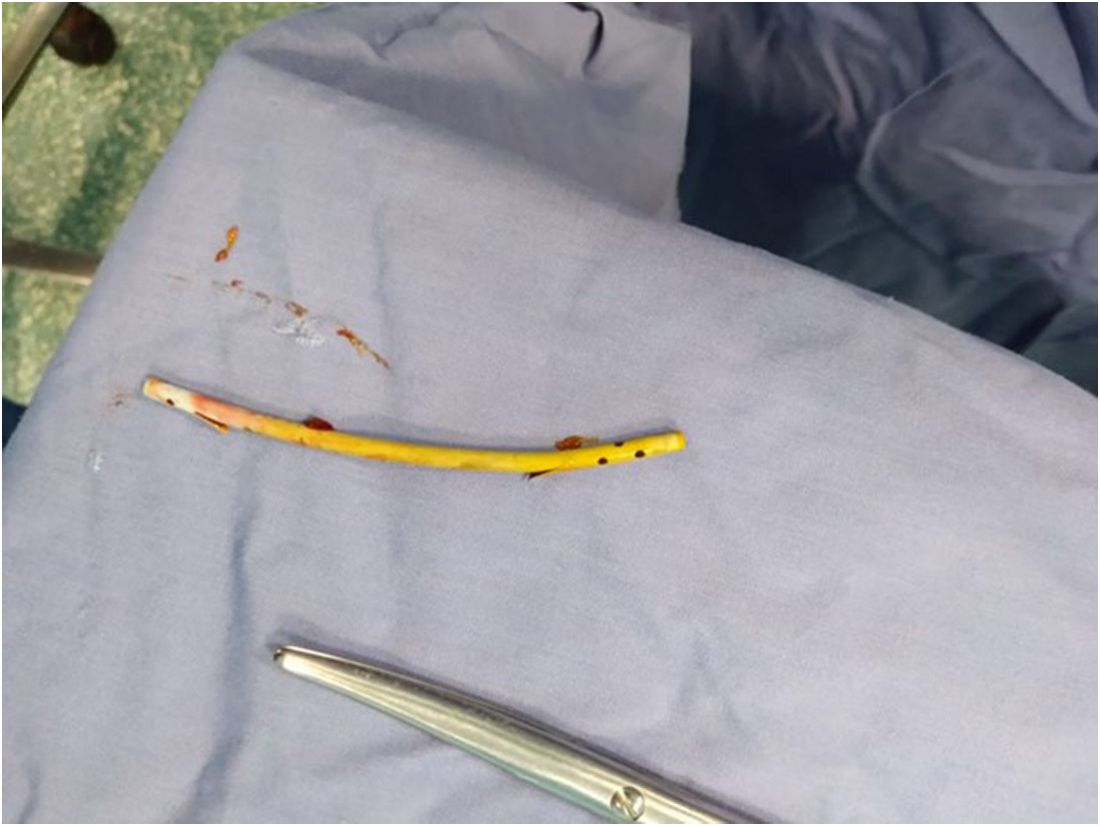
Plastic stent 6 cm in length with 10 Fr.

**FIGURE 2:**
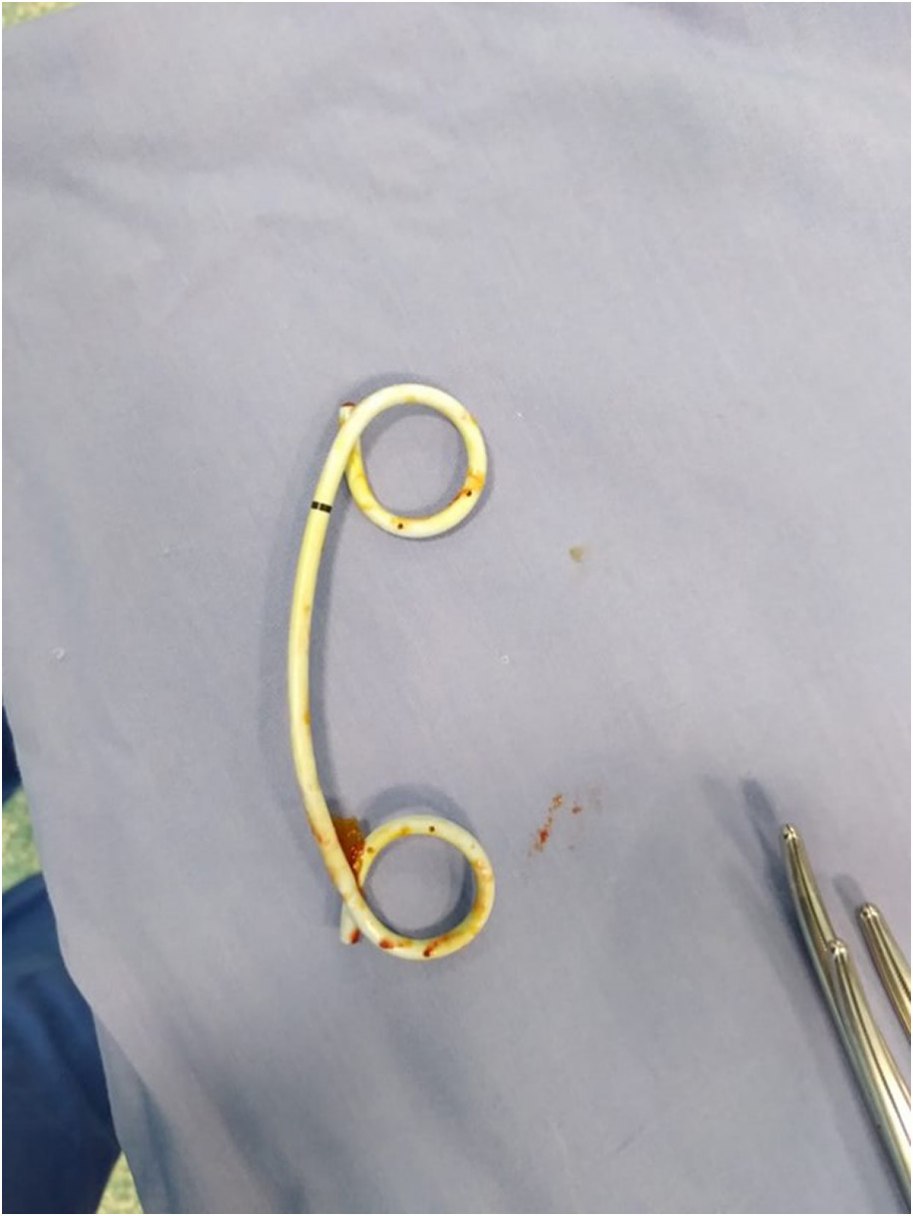
Double pigtail plastic stent 6 cm in length with 10 Fr.

She has a history of three admissions as a case of obstructive jaundice for which ERCP was performed four times, including one unsuccessful and three successful ERCPs with the placement of two types of plastic biliary stents in the last 7 months, as the following:

On August 4, 2020, she first showed up with hyperbilirubinemia and obstructive jaundice symptoms. After 2 days, an ultrasound taken at the time revealed a dilated CBD measuring 12 mm. Her first diagnostic ERCP was unsuccessful as it could not cannulate through the orifice and the precut incision because of respiration and peristalsis that hyoscine butyl bromide, glucagon, or sedatives could not stop. Still, it did reveal a protruding major duodenal papilla. Three days later, she underwent a second repeated diagnostic ERCP on August 9, and it was discovered that the CBD end had a benign stricture. On August 16, a plastic stent with a 6 cm 10 French (Fr) external (Figure 1) and a single internal flap was implanted following a third ERCP. A fourth ERCP was required to diagnose the issue because obstructive jaundice continued and recurred for months after that third ERCP with similar plastic stent replacement was performed. At that point, choledocholithiasis was discovered and believed to cause jaundice, with a dilated CBD reaching 14 mm and a smooth tapering end. As a result, a new double pigtail plastic stent (10 Fr 6 cm in length) was inserted (Figure 2). She also had a computed tomography scan of her abdomen and pelvis, which revealed signs of migrating stents. She was hospitalized for an urgent laparotomy in April 2021.

### 2.4 Past and surgical history

She had an open cholecystectomy 40 years ago, an open hysterectomy because of an unusual vaginal hemorrhage 35 years ago, and adhesiolysis because of a small intestine obstruction 30 years ago. These previous surgical procedures were substantial.

### 2.5 Examination

Upon assessment, she displays a low‐grade temperature, slight tachycardia, normal oxygen saturation, and normal pressure. There were three stars: a Pfannenstiel scar, a midline laparotomy scar, and a right subcostal scar. In addition, her abdomen was swollen, painful, tympanic to percussion, showing indications of peritonitis, and she could not urinate. A little hernia of the umbilicus that contains omental fat was also observed.

### 2.6 Laboratory examinations

From laboratory evaluation, the patient had WBC 12.3 k/μL and total bilirubin 10.3 mg/dL.

### 2.7 Imaging examinations

**FIGURE 3:**
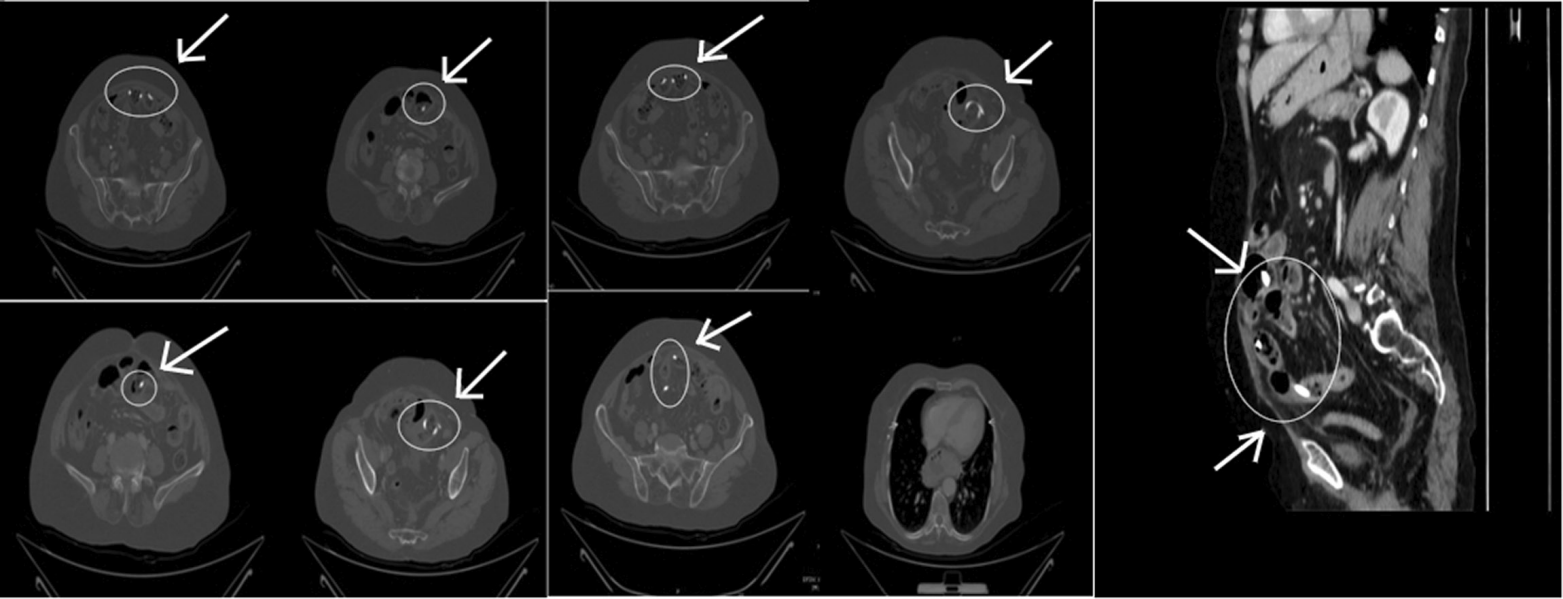
Computed tomography scan; a multiple serial axial and reformatted sagittal images showing the findings.

Computed tomography scan of the abdomen and pelvis with intravenous contrast provided multiple serial axials, reformatted sagittal, and coronal images (Figure 3). Evidence of at least two migrated biliary stents was found in the proximal and mid‐ileal loops, with one of them protruding into the intraperitoneal cavity and causing a localized perforation with small bowel ileus and peritonitis along with a few small foci of pneumoperitoneum, thickening of the circumferential wall surrounding it, and dilatation of the adjacent bowel loop, fat stranding, and high attenuation interloop free fluid.

Small amounts of free fluid and generalized mesenteric fat stranding were present in the pelvis and abdomen, consistent with peritonitis. Moreover, a simple diverticulum was seen in the sigmoid colon, and phleboliths were visible in the pelvis.

There was evidence of pneumobilia, intra‐ and extrahepatic biliary dilatation, and left sidedness of the hepatic ducts and CBD. In addition, the non‐visualization of the gallbladder is consistent with the patient's history of cholecystectomy. The liver, pancreas, and adrenal glands, which had normal attenuation, size, and enhancement, showed no apparent localized pathology. Bilaterally, there were a few small renal cortical cysts; the largest was in the left kidney's lower pole and measured about 0.8 × 1 cm. Other than that, there was no evidence of hydronephrosis, and both kidneys seemed to improve after intravenous contrast. Additionally, cystocele (the prolapse of the bladder into the vagina) and microcalcifications in the urinary bladder wall were seen. The patient's surgical history of bilateral salpingo‐oophorectomy and hysterectomy was compatible with the absence of the uterus and both ovaries.

There are prominent lymph nodes in the gastrohepatic, porta hepatic, para‐aortic, paracaval, common iliac, external, and internal iliac, and mesenteric regions. The largest lymph node measures about 6 mm in its short axis in the right common iliac region. Additionally, atherosclerotic changes can be seen in the abdominal aorta and its major branches.

BMD measurements show a decline in the investigated bone's bone density. Hemangiomas and multilayer anterior/posterior osteophyte complexes among the degenerative changes in the examined spine.

Regarding chest imaging, the region of the lung bases that could be seen had bi‐basal atelectatic changes, and both lung bases exhibit a mosaic attenuation pattern. In addition, small pleural‐based lung nodules were present in both lungs; the largest is atelectatic and measures about 3 millimeters. Finally, a sizable sliding hiatal hernia was compressing the IVC and heart.

### 2.8 Treatment

**FIGURE 4:**
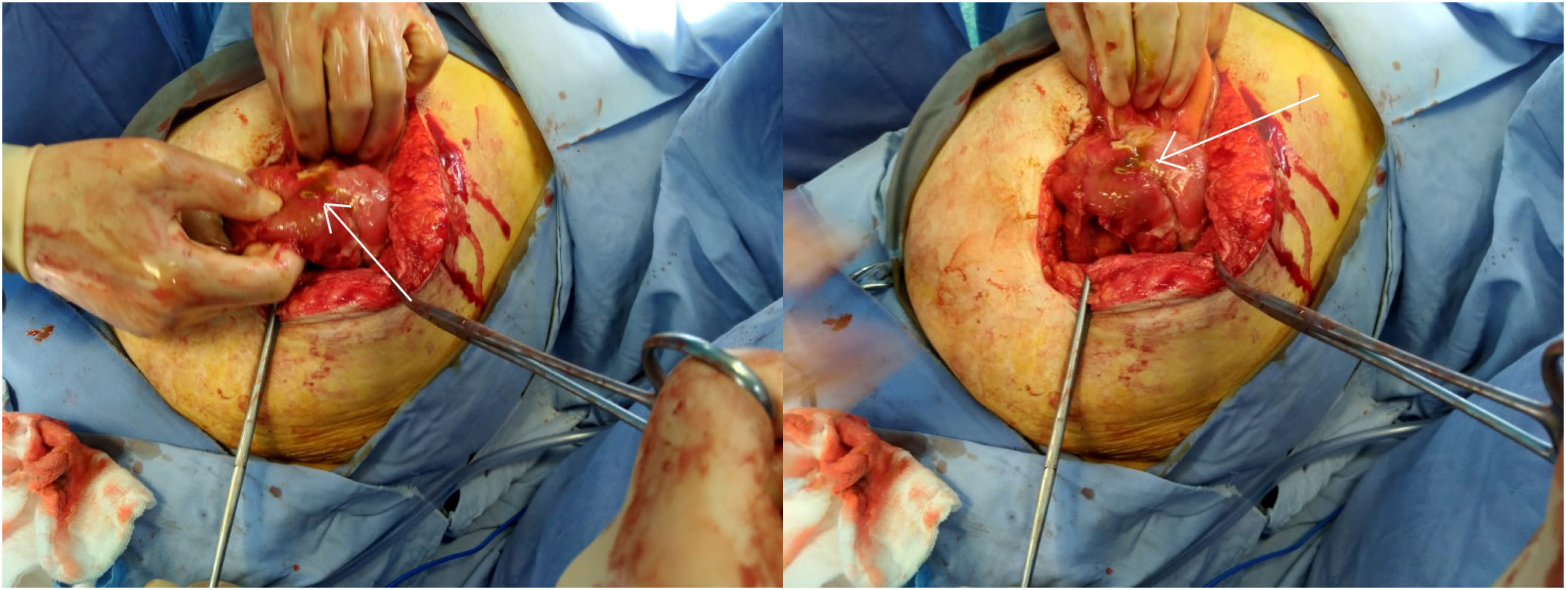
Intraoperative images demonstrating biliary stents perforation.

A doubly migrated biliary stent was the cause of the final diagnosis, which was distal small bowel perforation. The surgical team decided on an emergency laparotomy after consulting with the patient, and it was carried out to reveal extensive adhesion between the small bowel and abdominal wall, perforation of the distal small bowel brought on by the penetration of the plastic stent, along with hypertrophic changes and necrotic tissue at the site of perforation. The proximal and mid‐ileal loops' stents were both removed. Due to the severe inflammation, it was decided to remove the portion of the bowel with the perforation. As a result, the affected bowel was resected using a tension‐free stapled anastomosis from side to side (Figure 4).

### 2.9 Outcome and follow‐up

Following surgery, everything went smoothly, and nothing complicated happened. The patient was treated in the ICU for 24 h before being moved to the floor and sent home on the sixth postoperative day.

## 3 DISCUSSION

A significant scientific development of contemporary medicine is the endoscopic implantation of stents in the CBD or pancreatic duct, a regularly used technique to treat benign or malignant biliary or pancreatic tract stenosis or obstruction. It was first introduced in 1980 as a replacement to surgical choledochoduodenostomy for high‐risk or inoperable cases to relieve the biliary system.^6^ As the initial description of endoscopic biliary stent implantation, the entire procedure and the available stents have been greatly improved. This technique's popularity is steadily rising because it is a less invasive treatment than surgery. However, serious problems can occur during or after endoscopic treatments like upper endoscopy and biliary tract cannulation, despite their obvious benefits and the significant advancements in this field.^3^ Applying a biliary stent can create issues, including stent occlusion from clogging, which may lead to cholecystitis or cholangitis, pancreatitis from duct manipulation, bleeding, stent fracture, and stent migration. As various institutes have varying levels of experience, varied equipment is accessible, and different etiologic grounds for the intervention, the overall rate of biliary stent problems differs among them.^7^

According to Arhan et al.^2^ the migration rate was 8.58% (proximal 4.58% and distal 4.00%). In benign biliary strictures (BBSs), migration occurred more frequently than in malignant ones (13.7% vs. 5.3%). When compared to patients with numerous stents (2.7%), the rate of stent migration in BBSs was higher in cases with one (19.3%) and two stents (20.9%). When compared to instances with one stent (3.0%) and those with multiple stents (0%) in malignant biliary strictures (MBSs), migration happened more frequently (10.9%) in cases with two stents. Long stents migrated more commonly distally (73%) and short stents more frequently proximally (77%) in the BBSs. In the BBSs, migration was more frequently proximal (73.3%) than distal (76.9%) in cases of proximal stricture and distal (73.3%) in cases of distal stricture. The more extensive diameter expansion of the biliary system concerning benign reasons and the quick reduction in inflammation following the stent explain the higher frequency of stent migration in benign disorders. However, it is stated that in malignant illnesses, the migration rate is low due to stent fixation brought on by tumor growth.^4^, ^8^, ^9^ Malignant strictures and broad, short stents have been linked to proximal migration of the stent, whereas benign strictures and ampullary stenosis have been linked to distal migration of the stent.^4^ Instead of using a single oversized stent, some writers advise using numerous smaller stents to limit the likelihood of stent migration.^10^ Regular sphincterotomy during biliary stenting is not advised because the valves and sphincter of Oddi tonus may help to avoid distal migration. Proximal and distal migration of biliary stents is an additional classification. Distally migrating stents often traverse the intestine without any problems.^1^, ^11^

In our case, she underwent an open cholecystectomy 40 years before and open hysterectomy due to an uncommon vaginal hemorrhage 35 years prior, and adhesion‐lysis due to a small intestine obstruction 30 years prior. The bowel was less mobile due to the patient's condition. Therefore, there was a greater possibility that the stent would be damaged and fail to pass. Most institutions have policies to guarantee that all patients with stents are called back for stent removal.^7^

Endoscopic retrograde cholangiopancreatography along with endoscopic sphincterotomy, balloon, and basket techniques is the primary approach for removing stones from the CBD. Even though ERCP has an 80%–85% success rate for extracting stones,^1^, ^2^ in about 10%–15% of cases, the stone cannot be eliminated due to factors such as its size (>15 mm), short distal CBD length (36 mm), narrow angle (135°), or anatomical difficulties caused by the stone's impact.^12^, ^13^

If an attempt to remove a CBD stone using ERCP fails, a temporary plastic stent can be inserted to assist with bile drainage, act as a temporary measure until more advanced procedures can be done, and reduce the impact of the stone.^13^ So, based on our case, it is the procedure of choice.

The patient had conducted four ERCP, three of which were conducted in 2020 and the last performed in 2021. The first one showed a major bulging papilla; however, due to failure to cannulate the ampulla through the orifice and precut incision due to breathing and peristalsis, which could not be abolished with both hyoscine butyl bromide, glucagon, and sedatives. Accordingly, an ERCP was conducted 3 days later. The new one showed a recent scar of the last needle incision in the major papilla, markedly dilated CBD, and dilated CHD and intrahepatic ducts. Accordingly, cannulation was performed through the orifice, a large sphincterotomy incision was done, and the above cholangiogram was obtained. Sweeping with balloon 13 mm and then 16 mm was done with rapid clearance of the biliary tract, and no stones or sludge was seen. A week later, ERCP was conducted in which cannulation was performed through the previous sphincterotomy with balloon and occlusion cholangiogram performed, which showed the previous finding, sweeping with the balloon 13 and then 16 mm performed with the passage of clear bile, no stones, or sludge, as obstruction orifice is suspected at the orifice, a stent 6 cm by 10 Fr inserted with free bile flow noted. After 3 months, a third ERCP with a similar plastic stent replacement was performed In the year later, an ERCP was conducted, which showed dilation reaching about 14 mm with smooth tapering down regarding the major papilla. Additionally, cannulation was performed with a balloon and wire, and the occlusion cholangiogram showed no stents and the above feature in the biliary tract. Furthermore, sweeping was conducted with the removal of sludge; however, a stone was felt to be present inferiorly; accordingly, an extension of the orifice was performed with sphincterotomy, which resulted in the removal of stones around 8 mm with a large number of sludges, a double pigtail plastic stent 6 cm in length with 10 Fr inserted with free bile drainage.

A major complication from a migrated stent is intestinal perforation, which can happen anywhere in the small or large intestine. There are very few incidences of small bowel perforation, and most published cases of bowel perforation from migrating biliary stents involve either duodenal or large intestinal perforation. Most individuals with perforation will exhibit widespread peritonitis and septic symptoms at presentation.^7^ The level of infection in our case was high, and there was a sign of peritonitis.

There is a growing body of knowledge on this subject, and many therapeutic modalities have been suggested. A case series of stent migration requiring surgical intervention was reported by Diller et al.^14^ in 2003. The stents ranged in size from 7 to 14 Fr, with lengths between 7 and 12 cm. Three patients received stents: two received polyurethanes, one had a Teflon stent, and the other received metallic stents. Four patients had an acute pancreatitis diagnosis, and the fifth patient underwent liver transplantation and was given a preventive stent. From surgical respiratory failure, one of those five patients passed away. In this study, 987 individuals had a stent migration rate of 3.7%. In their literature analysis with 12 instances total, including 7 cases from 2000, Namdar et al.^1^; one described a rectal perforation caused by a migrated biliary stent. According to several studies, downstream migration occurs more frequently in benign biliary illness than in malignant biliary disease; this may be because the stenosis resolves when the inflammation subsides.^1^ In addition, they advise that regardless of the patient's clinical condition, any biliary stent that has shifted should be removed right away.^1^ Most of the early increasing corpus of research on endoscopic procedures for treating bowel perforation caused by migrating stents focuses on duodenal or distal large intestine perforation. Recently, Bureau et al.^15^ published a case series of six patients with over‐the‐scope clip treatment for lateral duodenal wall perforation caused by shifted plastic biliary stent. The endoscopic method could have been more practical since the bowel hole was in the middle of the jejunal loop. Additionally, axial images of the abdomen and pelvic X‐ray were conducted with oral and intravenous contrast, which showed at least three migrated stents seen in the proximal and mild ileal loops, one of them seen in the left lower quadrant protruding into the intraperitoneal cavity‐causing localized perforation associated with bowel loop, fat, stranding and high attenuation interloop free fluid, finding concerning for biliary stent migration causing localized small bowel perforation with small bowel ileus and peritonitis.

Which made us worry that an endoscopic mucosal repair might not be durable. As a result, we went straight to surgery.

In a systematic analysis of the literature from 2000 to October 2022, we identified 85 cases of intestinal perforation caused by migrating biliary stents (Table 1). The following search algorithm was used to find the appropriate articles in the MEDLINE bibliographical database (latest search: October 3, 2022), it was adapted from Zorbas et al.^7^ algorithm used in their recently published paper and a continuation of their work to update the literature: ((“intestinal perforation” [MeSH Terms] OR (“intestinal” [All Fields] AND “perforation” [All Fields]) OR “intestinal perforation” [All Fields] OR (“bowel” [All Fields] AND “perforation” [All Fields]) OR “bowel perforation” [All Fields]) AND (“migrate” [All Fields] OR “migrated” [All Fields] OR “migrates” [All Fields] OR “migrating” [All Fields] OR “migration” [All Fields] OR “migrational” [All Fields] OR “migrations” [All Fields] OR “migrator” [All Fields] OR “migrators” [All Fields]) AND “biliary” [All Fields] AND (“stent s” [All Fields] OR “stentings” [All Fields] OR “stents” [MeSH Terms] OR “stents” [All Fields] OR “stent” [All Fields] OR “stented” [All Fields] OR “stenting” [All Fields])) AND (2000:2022 [pdat]). The references of relevant articles were further searched, and publications connected to our topic were included. E‐Videos, E‐pictures, and manuscripts in languages other than English were not included. Manuscripts with full text readily available online were used. Additionally, cases were disregarded if the whole text was not accessible online.

**TABLE 1:** Summary of the systematic literature analysis (2000–2022).

No	Year	Age, years	Gender	Type of stent^a^	Site of perforation	Treatment	Country	Mortality	Stent length	Stent size	Refs.
1	2000	81	M	P	SB	ST	Norway	Y	6.5	10 Fr	^16^
2	2000	86	M	P	LB	ST	Norway	N	5	7 Fr	^16^
3	2000	74	M	P	DU	ET	Spain	N	15	10 Fr	^17^
4	2001	58	M	P	DU	ET	Italy	N	12	10 Fr	^18^
5	2001	43	F	P	DU	ET	India	N	NA	10 Fr	^19^
6	2001	NA	NA	P	SB	ST	United States	N	12	11.5 Fr	^20^
7	2001	88	F	P	DU	ST	Germany	N	10	7 Fr	^21^
9	2001	31	F	NA	BD	ST	Denmark	N	NA	NA	^8^
10	2001	47	M	P	LB	ST	Spain	N	10	10 Fr	^22^
11	2002	72	F	P	SB	ST	Italy	N	NA	12 Fr	^23^
12	2002	NA	NA	P	SB	ST	United States	N	7	8.5 Fr	^24^
13	2003	85	F	P	LB	ST	Germany	N	NA	NA	^25^
14	2003	86	M	P	DU	ET	Italy	Y	15	10 Fr	^26^
15	2003	27	F	P	SB	ST	Germany	N	12	12 Fr	^11^
16	2003	58	M	P	LB	ET‐ST	Germany	N	10	7 Fr	^11^
17	2003	60	F	P	SB	ST	Germany	N	12	14 Fr	^11^
18	2003	64	M	M	LB	ST	Germany	Y	7	10 Fr	^11^
19	2003	65	M	M	NA	ST	Germany	N	7	10 Fr	^11^
20	2003	62	F	P	LB	ST	Argentina	N	NA	8 Fr	^27^
21	2003	62	F	P	SB	ST	Argentina	N	NA	5.5/10 Fr	^27^
22	2003	80	F	P	LB	ST	Australia	N	10	10 Fr	^28^
23	2004	65	F	P	LB	ST	United States	N	NA	NA	^29^
24	2005	69	M	M	DU	ST	United States	N	NA	NA	^30^
25	2006	55	M	P	DU	ET	Greece	Y	NA	NA	^31^
26	2006	74	M	P	DU	ST	India	NA	10	7 Fr	^32^
27	2006	54	F	P	SB	ST	United Kingdom	N	7	10 Fr	^33^
28	2006	85	M	P	DU	ST	Italy	N	10	9 Fr	^34^
29	2007	65	F	P	LB	ST	Germany	N	10	12 Fr	^1^
30	2008	75	M	P	DU	ST	Taiwan	N	NA	NA	^35^
31	2008	52	F	P	DU	ST	Turkey	N	10	8.5 Fr	^36^
32	2008	67	M	P	DU	ST	Australia	Y	NA	5/10 Fr	^37^
33	2008	43	M	P	DU	ET	Belgium	N	NA	NA	^38^
34	2008	71	F	P	SB	ST	Belgium	N	NA	NA	^38^
35	2009	77	M	P	LB	PI	United States	N	12	10 Fr	^39^
36	2009	76	F	P	SB	PI	United States	N	NA	10 Fr	^40^
37	2009	59	F	P	SB	ST	Turkey	N	7	11 Fr	^41^
38	2011	58	M	P	DU	PI	United Kingdom	N	10	8.5 Fr	^42^
39	2011	65	F	P	LB	ST	Germany	N	10	10 F Fr	^43^
40	2011	73	NA	P	LB	ST	France	N	5	10 Fr	^44^
41	2011	75	M	P	SB	ST	United Kingdom	N	NA	NA	^45^
42	2011	70	M	P	DU	ET	China	N	NA	8.5 Fr	^46^
43	2011	82	F	P	LB	ET	United Kingdom	N	7	7 Fr	^47^
44	2012	55	M	P	DU	ET	South Korea	N	7/5	5 Fr	^48^
45	2012	27	F	P	DU	ST	United Kingdom	N	12	7 Fr	^49^
46	2012	87	F	P	DU	ET	United States	N	15	8.5 Fr	^50^
47	2012	73	M	P	LB	ET	Spain	N	12	10 Fr	^51^
48	2012	50	NA	P	LB	ET	Belgium	N	NA	NA	^52^
49	2013	51	M	P	DU	ST	S. Arabia	N	10	10 Fr	^53^
50	2013	66	M	P	LB	ET	United Kingdom	N	NA	NA	^54^
51	2013	50	M	M	SB	ST	India	N	NA	NA	^55^
52	2014	67	M	P	DU	ST	United States	Y	12	10 Fr	^56^
53	2014	73	M	P	LB	ST	Australia	N	5	10 Fr	^57^
54	2014	66	F	P	DU	ET	The Netherlands	N	15	NA	^58^
55	2015	48	M	P	DU	ET	United States	N	NA	NA	^59^
56	2015	NA	F	P	LB	ST	Italy	N	12	12 Fr	^60^
57	2015	NA	F	P	LB	ET	Italy	N	12	12 Fr	^60^
58	2015	52	F	P	SB	ST	Turkey	N	NA	NA	^61^
59	2015	NA	M	P	LB	ST	United Kingdom	Y	NA	NA	^62^
60	2016	85	F	P	SB	N.A.	Turkey	Y	NA	NA	^3^
61	2017	75	F	P	LB	ST	Greece	N	NA	NA	^63^
62	2018	57	M	P	DU	ET	United States	N	15	8.5 Fr	^64^
63	2018	79	F	P	DU	ET	United States	N	12 + 15	7 + 10 Fr	^65^
64	2018	87	M	P	DU	ST	Greece	N	15	10 Fr	^66^
65	2018	20	M	P	SB	ST	Turkey	N	NA	NA	^67^
66	2019	71	M	P	DU	ET	France	N	12	8.5 Fr	^68^
67	2019	50	M	P	DU	ET	South Korea	N	10	10 F	^69^
68	2019	78	M	P	DU	ET	South Korea	N	10	7 Fr	^69^
69	2019	72	M	P	DU	ET	South Korea	N	12	10 Fr	^69^
70	2019	84	F	P	DU	ET	South Korea	N	12	10 Fr	^69^
71	2019	73	F	P	DU	ET	South Korea	N	15	10 Fr	^69^
72	2019	63	F	P	DU	ST	Jordan	N	10	10 Fr	^70^
73	2019	65	F	P	LB	ST	Portugal	N	5	10 Fr	^71^
74	2019	79	F	P	LB	ST	United States	N	10	7 + 10 Fr	^72^
75	2020	90	F	P	SB	ST	Australia	N	9	10 Fr	^73^
76	2020	84	F	P	SB	ST	Australia	N	7	10 Fr	^74^
77	2020	72	M	P	DU	ET	China	N	9	8.5 Fr	^5^
78	2020	84	M	P	DU	ET	China	N	12	7 Fr	^5^
79	2020	52	M	P	DU	ET	China	N	9	8.5 Fr	^5^
80	2021	80	F	P	JU	ST	Australia	N	NA	10 Fr	^75^
81	2021	74	M	P	LB	ST	Republic of Korea	N	7	10 Fr	^76^
82	2018	66	F	P	AP	ST	Finland	N	5	10 Fr	^77^
83	2020	54	F	P	IL + JU	ST	United States	N	7 + 7	7 Fr + 8.5 Fr	^7^
84	2020	54	M	P	LB	ST	China	N	NA	NA	^78^
85	2022	33	M	P	DU	ST	Nepal	N	NA	NA	^79^

Abbreviations: AP, appendix; BD, bile duct; DU, duodenum; ET, endoscopic treatment; IL, ileum; JU, jejunum; LB, large bowel; M, metallic; NA, not available; PI, percutaneous intervention; P, plastic; SB, small bowel; ST, surgical treatment.

^a^ Time interval from stent placement to complication in days.

Our literature review found that 85 patients had similar cases in which the mean age was (65.63 ± 15.63), 50.0% were male patients, 45.2% were female, and the rest were missing data. 94.0% of the cases were plastic stents, whereas the rest were metallic, 4.8%, and one missing data. Regarding the stent length, it had a range of 10 (15–5) cm, and most patients had either a 10 or 12‐cm stent (50.8%). On the other hand, regarding the stent size, it had a range of 9 (14–5) Fr. Moreover, duodenum was the site to get perforated mostly in the reported cases, 36 (42.4%). Then, large bowels, 25 (29.4%), and small bowels, 18 (21.2%). Surgical treatment was used more in the previous cases 53 (63.1%) than Endoscopic treatment 28 (33.3%). Finally, regarding mortality, only 8 people (9.5%) died, while 75 (89.3%) survived.

## 4 CONCLUSIONS

The duodenum is the organ most commonly affected by perforation, and the most frequent type of stent that causes bowel perforation is plastic stents. Abdominal CT scan is the most useful imaging modality for detection of migrated stents and associated complications and the usual treatment for this is to remove the stent surgically, but some research suggests that endoscopic removal and mucosal healing may also be possible in some instances. The middle of the gut has yet to be fully explored and may be an area for further research. The endoscopic implantation of biliary stents is a practical and efficient method for temporarily relieving pressure in the biliary system. According to the literature, the primary cause of perforation is extensive adhesion. When using stents for long‐term therapy, it is essential to consider the possibility of stent migration, which can lead to life‐threatening complications. It can be challenging to make a correct diagnosis because there are no typical symptoms. More measures should be considered in the future prevention of stent migration and perforation in ERCP which involves careful patient selection, appropriate indications and techniques, optimal stent choice and placement, and regular follow‐up with timely stent removal when needed.

## AUTHOR CONTRIBUTIONS

**Ibraheem M. Alkhawaldeh:** Writing – original draft; writing – review and editing. **Osama Shattarah:** Writing – original draft; writing – review and editing. **Jehad F AlSamhori:** Writing – original draft; writing – review and editing. **Mohammad Abu‐Jeyyab:** Writing – original draft; writing – review and editing. **Abdulqadir J Nashwan:** Writing – original draft; writing – review and editing.

## FUNDING INFORMATION

None.

## CONFLICT OF INTEREST STATEMENT

All authors declared no conflict of interest.

## CONSENT

Written informed consent was obtained from the patient to publish this report in accordance with the journal's patient consent policy.

## Data Availability

All data generated or analyzed during this study are included in this published article.
